# Factors Associated with the Prescribing of High-Intensity Statins

**DOI:** 10.3390/jcm9123850

**Published:** 2020-11-27

**Authors:** Armando Chaure-Pardos, Sara Malo, María José Rabanaque, Federico Arribas, Belén Moreno-Franco, Isabel Aguilar-Palacio

**Affiliations:** 1 Department of Preventive Medicine and Public Health, University of Zaragoza, 50009 Zaragoza, Spain; achaure@salud.aragon.es (A.C.-P.); rabanake@unizar.es (M.J.R.); farribas@aragon.es (F.A.); mbmoreno@posta.unizar.es (B.M.-F.); iaguilar@unizar.es (I.A.-P.); 2 Fundación Instituto de Investigación Sanitaria de Aragón (IIS Aragón), 50009 Zaragoza, Spain; 3 GRISSA Research Group, 50009 Zaragoza, Spain

**Keywords:** cardiovascular diseases, prevention and control, healthy lifestyle, hydroxymethylglutaryl-coa reductase inhibitors, practice patterns, physicians’

## Abstract

In this study, we investigated the relationship between sociodemographic, clinical, anthropometric, and lifestyle characteristics and the type of statin prescribed for primary prevention of cardiovascular disease (CVD). We conducted an observational study in workers who began statin treatment. Statin therapy was categorized as “high-intensity” or “low–moderate-intensity”. Workers were classified according to the alignment of their statin therapy with the recommended management practices. Logistic regression models were used to evaluate the association between the different variables studied and the probability of being prescribed high-intensity statins. The only variables associated with a higher probability of being treated with high-intensity statins were increased physical activity (>40 versus <20 METs (metabolic equivalent of task) h/wk; odds ratio (OR), 1.65; 95%CI, 1.08–2.50) and, in diabetics, higher low-density lipoprotein cholesterol (LDL-C) levels (≥155 mg/dL versus <155 mg/dL; OR, 4.96; 95%CI, 1.29–19.10). The model that best predicted treatment intensity included LDL-C, diabetes, hypertension, smoking, and age (area under the Receiver Operating Characteristic curve (AUC), 0.620; 95%CI, 0.574–0.666). The prescribing and type of statin used in primary CVD prevention did not correspond with the indications in current guidelines. The probability of receiving high-intensity statins was higher in diabetics with high LDL-C levels and in more physically active individuals. These findings underscore the great variability and uncertainty in the prescribing of statins.

## 1. Introduction

Clinical practice guidelines to prevent cardiovascular disease (CVD) are based on risk assessment, recommendation of a healthy lifestyle, and, in some cases, pharmacological treatment, including lipid-lowering therapy [1,2]. Current European guidelines for CVD prevention consider statins as a first-line lipid-lowering option [1]. These drugs have proven efficacy in the primary prevention of CVD, reducing morbidity and mortality in individuals with moderate and high risk [3]. However, their effectiveness in low-risk individuals remains a topic of debate [4,5].

According to their ability to lower blood levels of low-density lipoprotein cholesterol (LDL-C), statins are typically classified as low-, moderate-, or high-intensity [6,7]. This classification depends on the drug and the dose used. Guidelines do not indicate the type of statin that should be prescribed, but establish target LDL-C levels that depend on the risk of CVD. Thus, when deciding the type of statin to be prescribed, the physician must be aware of the patient’s CVD risk, as well as current and target LDL-C levels.

Given these rather nonspecific recommendations, it is of interest to know which factors (including classical CVD risk factors, basal CVD risk, and lifestyle-related factors) are considered most important in clinical practice when prescribing statins. This knowledge can be useful to guide prescription and to adjust it to established standards. Some studies have described the characteristics of statin users without stratifying by statin intensity [8,9]. Others that have collected information from administrative databases benefit from large sample sizes, but tend to include incomplete clinical data and lack information on patient lifestyles [10]. Finally, some authors have focused their analyses on individuals already in treatment, neglecting new users and therefore not considering the characteristics of the user at the moment of the decision to treat [11].

The aim of the present study was to characterize the profile of new users of statins for primary CVD prevention in a cohort of workers, and to investigate the association between these characteristics and the type of statin prescribed.

## 2. Experimental Section

### 2.1. Study Design and Participants

This study was based on the Aragon Worker’s Health Study cohort (AWHS). The AWHS is a longitudinal prospective cohort study that was designed to evaluate the evolution of traditional and emergent CVD risk factors and their association with the prevalence and progression of subclinical atherosclerosis in a population of middle-aged car plant workers in Spain [12]. Recruitment was carried out for the most part in 2009 and 2010, and continues today. From this population, a subgroup of 2667 workers who were aged 40–55 years at baseline was selected. This subgroup completed additional questionnaires about cardiovascular and lifestyle factors, including diet, physical activity, and sleep habits. The workers were also invited to undergo a noninvasive imaging examination for subclinical atherosclerosis.

For the present study, we selected from the aforementioned subgroup workers who began statin therapy for primary prevention of CVD between 1 July 2010 and 31 December 2018. New users were defined as those who did not receive a prescription for statins during the preceding 6 months. Workers in treatment for primary prevention of CVD were those with no recorded hospital admission for CVD in the hospitalization database of the Aragon Health System preceding their first statin prescription. Patients with CVD were defined as those with a main diagnosis corresponding to any of the following International Classification of Diseases 10th revision (ICD-10) codes: G45, G46, G81–G83, I20–I28, I46, I49.0, I50, and I60–I79. 

### 2.2. Data Sources and Variables

Information on statin treatment was obtained from the Farmasalud database, which collects information on drugs dispensed by pharmacies and funded by the Aragon Health System. Drugs were classified according to the 2019 ATC/DDD (Anatomical Therapeutic Chemical Classification System/Defined Daily Dose) system, defined by the World Health Organization. We identified workers with at least one prescription during the study period of a drug corresponding to the following ATC codes: C10AA (hydroxymethylglutaryl-CoA (HMG CoA) reductase inhibitors), C10BA (HMG CoA reductase inhibitors in combination with other lipid-modifying agents), C10BX (HMG CoA reductase inhibitors, other combinations). 

Information on other CVD treatments was also collected. Agents corresponding to the following ATC codes were classified as antihypertensives: C02 (antihypertensives), C03 (diuretics), C07 (beta-blocking agents), C08 (calcium channel blockers), and C09 (agents acting on the renin–angiotensin system). Prescriptions for the treatment of diabetes were defined as those corresponding to ATC code A10 (drugs used in diabetes). The following criterion was applied to define individuals receiving co-treatments: the existence of at least one drug prescription corresponding to the aforementioned codes during the year preceding the first statin prescription. Given that diuretics and beta-blocking agents are drugs also prescribed for other indications, prescribing of these drugs was only considered co-treatment if the patient received at least three distinct prescriptions within the same year. We considered that this frequency was indicative of regular use. Even so, to assess the possible bias of this assumption, we performed sensitivity analyses: (i) considering them as co-treatment if the patient received at least one prescription in the same year; (ii) not considering them as co-treatment at all.

In addition to information on other CVD preventive treatments, for all participants in the present study, we extracted information on working characteristics, clinical and anthropometric measurements, smoking status, and diagnoses of other diseases that may modify CVD risk. For these parameters, we selected for each worker data collected as close as possible before the date of the first statin prescription. 

Data pertaining to each individual work situation included the number of years working in the factory, work type, and work shift. Work type was classified as manual or sedentary. Work shifts in the plant were classified as rotating morning/afternoon shift, rotating morning/afternoon/night shift, central shift, and night shift. 

For clinical and anthropometric characterization, we extracted the following data, which were recorded in annual medical exams as described in Casasnovas et al. [12]: height, weight, serum creatinine, total and high-density lipoprotein (HDL)-cholesterol, triglycerides, serum glucose, whole blood glycosylated hemoglobin (HbA1c), and blood pressure. Annual data on smoking status were also extracted. 

Diseases considered to modify the risk of CVD were rheumatoid arthritis (RA), arrhythmias, and transient ischemic accident. Diagnoses corresponding to these diseases (International Classification of Primary Care codes L88, K80, and K89, respectively) were extracted from the Aragon primary care database.

Lifestyle of the study participants was characterized based on alcohol intake, adherence to a Mediterranean diet, physical activity, sleep, and sedentary time. A semiquantitative food-frequency questionnaire previously validated in Spain [13] was used to assess dietary habits. Leisure time physical activity was assessed with the validated Spanish version [14] of the physical activity questionnaire used in the Nurses’ Health Study and Health Professionals’ Follow-up Study. To estimate sedentary time, we included the number of hours (from “never” to “9 or more than 9 h a day”) spent sitting, as reported by the participant, during both working and leisure time in a typical weekday. The same procedure was followed to collect information on the number of hours spent sleeping per night.

The presence of subclinical atherosclerosis in both carotid and femoral arteries was determined by ultrasound, according to the procedures described in Laclaustra et al. [15]. Plaque was defined as a focal structure protruding ≥ 0.5 mm into the lumen or reaching a thickness ≥ 50% of the surrounding intima. 

### 2.3. Analyses

Statin therapy was categorized as “high-intensity” (atorvastatin or rosuvastatin) or “low–moderate-intensity” (simvastatin, lovastatin, pravastatin, fluvastatin, or pitavastatin) [7].

Clinical and anthropometric variables were defined as follows. Glomerular filtration rate (eGFR) was estimated using the modification of diet in renal disease (MDRD) formula [16], and an eGFR < 60 mL/min/1.73 m^2^ was considered indicative of chronic kidney disease [17]. Low-density lipoprotein cholesterol (LDL-c) was estimated using Friedewald’s estimation [18]. Applying the risk factor definitions proposed by the 2016 European guidelines on CVD prevention in clinical practice [1], we defined a worker as diabetic if he was treated with antidiabetics, had a HbA1c ≥ 6.5%, or had fasting plasma glucose ≥ 126 mg/dL. Criteria for hypertension were systolic blood pressure ≥ 140 mmHg, diastolic blood pressure ≥ 90 mmHg, or existence of a prescription for antihypertensive treatment. To assess adherence to a Mediterranean diet, we used the Alternate Mediterranean Dietary Index (aMED) [19]. To determine the total physical activity performed by each worker, METs (metabolic equivalent of task) were assigned for each activity [20] and multiplied by the number of hours per week that the worker dedicated to the activity. The total amount of MET-h/week was calculated as the sum of the MET-h of the different activities. We used 5.5 h/day as a cut-off point for sedentary time, following previous findings of increased body mass index (BMI), waist circumference, and insulin levels in this same cohort [21]. According to sleeping time, workers were divided into two groups: <6 h/night or ≥6 h/night. This decision was based on the lower limit in the National Sleep Foundation’s recommendations for adults [22]. 

Finally, we calculated the 10 year risk of a first fatal atherosclerotic event using the Systemic Coronary Risk Estimation (SCORE) for low-risk countries [23]. Next, participants were classified according to European guidelines [1] as low, moderate, high, or very high risk by combining their SCORE and other CVD risk factors including diabetes, chronic kidney disease, and extreme LDL-C or blood pressure values. 

Based on this risk classification and blood LDL-C levels, European guidelines recommend certain target LDL-C levels that should be achieved to reduce CVD risk. Following these guidelines, we classified workers according to the recommended management practices as follows: “statins not indicated” if, based on CVD risk and LDL-C levels, only lifestyle advice is recommended; “LDL-C reduction < 50%” if drug treatment is indicated and LDL-C should be reduced by less than 50% to reach the target LDL-C levels; “LDL-C reduction ≥ 50%” if drug treatment is indicated and LDL-C should be reduced by at least 50% to reach target levels.

All variables were described for the global population and stratified according to statin intensity. For each variable, we performed statistical analyses to assess the difference between treatment intensity groups. The Student’s t-test was used for quantitative variables. The chi-squared test was used to analyze categorical variables when the number of workers exceeded 30. Low-frequency variables were analyzed using Fisher’s exact test.

To evaluate the association between the different factors studied (sociodemographic, clinical, and lifestyle) and the probability of being prescribed high-intensity versus low–moderate-intensity statins, we used logistic regression models, with statin intensity as a dependent variable. First, we computed unadjusted models. Next, we developed multivariable models that included traditional CVD risk factors and variables for which we obtained a *p*-value < 0.1 in the unadjusted analysis. We also included interaction terms in the model. For inclusion in the models, continuous variables were categorized using pre-established cut-off points. Since we did not know how these variables would behave in the model, we established as a reference the category that allowed us to find significant differences between groups or, failing that, the lowest category.

Finally, to assess the ability of CVD risk factors to predict the prescribing of high-intensity statins, we developed logistic regression models. Explanatory variables were incorporated sequentially according to the magnitude of their association in the previous multivariable models. Predictive power was evaluated by calculating the area under the receiver operating characteristic (ROC) curve (AUC).

All analyses were performed using STATA version 14 (StataCorp, College Station, TX, USA).

### 2.4. Ethical Issues

All subjects gave their informed consent for inclusion before they participated in the study. The study was conducted in accordance with the Declaration of Helsinki, and the protocol was approved by the Ethics Committee of Aragon (Project identification code PI17/00042).

## 3. Results

A total of 683 workers began treatment with statins during the follow-up. Table 1 shows their sociodemographic, clinical, and anthropometric characteristics. The mean age of new statin users was 53 years, 5% were women, and most performed manual work with rotating shifts. More than half were classified as having hypertension, 81% were overweight or obese, and 10% had diabetes. Table 2 shows lifestyle and imaging characteristics. More than a third of the new statin users smoked at the time of prescription, 15% consumed more than 40 g of alcohol per day, and 36% had low adherence to a Mediterranean diet. More than half sat for at least 5.5 h a day, and 20% slept less than 6 h per night. Subclinical atherosclerosis was more frequent in the femoral artery than in the carotid artery.

High-intensity statins were prescribed to 323 (47%) of the new statin users. Workers prescribed low–moderate-intensity and high-intensity statins had similar characteristics. Mean cholesterol and LDL-C levels were higher in workers prescribed high-intensity statins, although these differences did not reach statistical significance. Subclinical atherosclerosis was more frequent in workers prescribed high-intensity statins.

**Figure 1:**
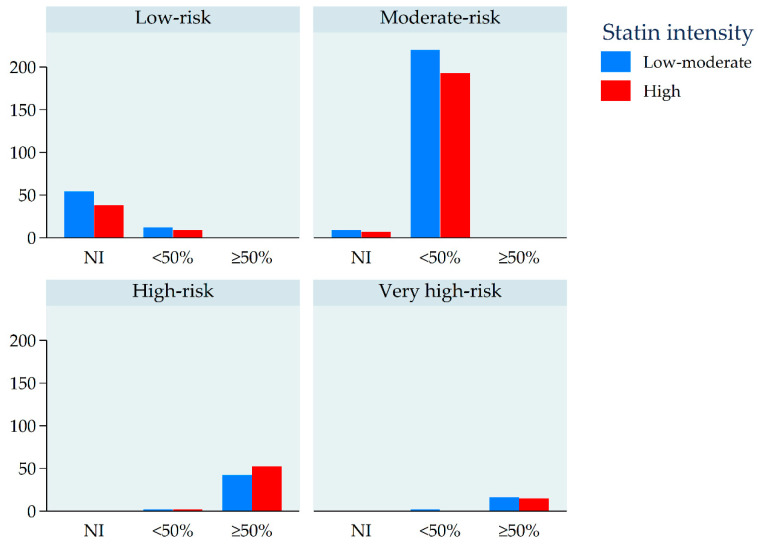
Number of workers prescribed low–moderate- or high-intensity statins, stratified by cardiovascular disease (CVD) risk and recommended management. CVD risk: classification of cardiovascular risk according to current European guidelines on CVD prevention, based on the combination of SCORE value and the presence of other CVD risk factors. Recommended management: recommended patient management according to current European guidelines on CVD prevention, based on CVD risk, current LDL-cholesterol (LDL-C) levels, and target LDL-C levels. NI: drug treatment “not indicated”, only lifestyle advice recommended. <50%: drug treatment indicated to reduce LDL-C by <50% to reach target LDL-C levels. ≥50%: drug treatment indicated to reduce LDL-C by ≥50% to reach target LDL-C levels.

Figure 1 shows the distribution of workers according to their CVD risk, management (as recommended by European guidelines), and statin treatment intensity. The largest group is that of workers with a moderate risk of CVD for whom pharmacological treatment is recommended to reduce LDL-C by less than 50%. In this group of 413 workers, 220 (53%) were prescribed low–moderate-intensity statins. The second-largest group is that of workers with a high risk of CVD for whom pharmacological treatment is recommended to reduce LDL-C by more than 50%. In this group of 94 workers, 42 (45%) were prescribed low–moderate-intensity statins. A total of 108 workers with low or moderate risk did not meet the criteria for pharmacological treatment. Of these, 45 (42%) were prescribed high-intensity statins.

Table 3 shows the results of unadjusted and multivariable logistic models to evaluate the association between cardiovascular risk factors and the prescribing of high- versus low–moderate-intensity statins. Given that we observed an interaction between LDL-C levels and diabetes in the context of prescribing of high-intensity statins, this interaction term was included in the models. Higher levels of physical activity (>40 versus <20 METs h/wk; OR, 1.65; 95%CI, 1.08–2.50) and, in diabetics, LDL-C levels ≥ 155 mg/dL versus < 155 mg/dL (OR, 4.96; 95%CI, 1.29–19.10) were associated with a higher probability of high-intensity statin prescribing. In non-diabetics, we observed no association between the prescribing of high-intensity statins and LDL-C levels. Similarly, there was no association detected between the prescribing of high-intensity statins and age, smoking status, or the presence of hypertension or diabetes.

**Figure 2:**
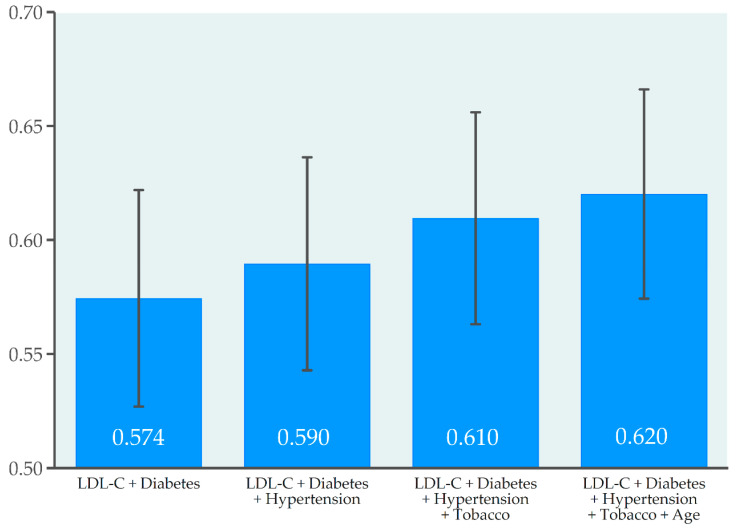
Prescribing of high-intensity statins: predictive capacity of clinical and analytical variables. Data are represented as the area under the receiver operating characteristic (ROC) curve and 95% confidence intervals (error bars). Abbreviation: LDL-C, low-density lipoprotein cholesterol.

We conducted predictive models in order to know which variables predict the prescribing of high-intensity statins. The best model obtained included the variables LDL-C, diabetes, hypertension, smoking, and age (AUC, 0.620; 95%CI, 0.574–0.666). Nonetheless, almost 40% of variability remained unexplained (Figure 2).

## 4. Discussion

The present study examines the sociodemographic, clinical, and lifestyle factors that determine the initial prescribing of high-intensity statins in clinical practice with the objective of achieving the LDL-C target levels recommended in European guidelines on CVD prevention. Our results indicate that while the choice of statin treatment intensity is associated with certain CVD risk factors, including LDL-C levels in diabetics, in a large proportion of cases it appears not to be based on the clinical guidelines criteria.

Low–moderate-intensity statins were prescribed slightly more than high-intensity statins, regardless of basal CVD risk and the need for LDL-C reduction to reach the target levels recommended in current guidelines. Thus, several workers who did not require a large decrease in LDL-C levels were prescribed high-intensity statins, and some workers who required LDL-C reductions of over 50% to reach target levels began treatment with low–moderate-intensity statins. Strikingly, more than 10% of workers included in our study began treatment with statins (almost half with high-intensity statins) despite the absence of any such indication in the European guidelines.

In our study population, the probability of receiving high-intensity statins was higher in workers with diabetes and high LDL-C levels, in those who performed more physical activity, and in those with subclinical atherosclerosis in the femoral artery. In fact, a greater proportion of workers being treated with high-intensity statins had carotid and femoral atherosclerosis, although these differences were not maintained after adjusting for other confounding variables.

Macías Saint-Gerons et al. [10] reported an association between very high LDL-C levels and the probability of prescribing high-intensity statins for primary CVD prevention. In that study, which included data from almost 70,000 first-time users between 2007 and 2011 in Spain, the authors also detected an association between the prescribing of high-intensity statins and male sex, high BMI, and smoking. Our study therefore supports a possible role of LDL-C levels, among others, in the decision to prescribe high-intensity statins. This makes sense since high-intensity statins reduce LDL-C levels to a greater extent than the others. On the other hand, diabetic patients have approximately twice the risk of CVD than non-diabetics [24]. In patients with this combination of risk factors, achieving lower LDL-C target levels is required [1]. An attempt to compensate their excess of CVD risk may be made by prescribing a high-intensity statin. Thus, our study results suggest that prescribers may be guided by simple indications, such as that the presence of the combination of high LDL-C + diabetes, which requires a more intense treatment.

In our cohort, high-intensity statins were also more frequently prescribed to workers who performed more physical activity. Ho et al. [11], using data from the Australian Diabetes, Obesity and Lifestyle Study, conducted a cross-sectional analysis of patients taking statins and found that those who performed insufficient physical activity were more likely to be taking high-intensity statins. Although our results differ with those of Ho et al., it should be noted that the latter study population consisted of individuals subjects who were already taking statins, while ours includes only workers who began this treatment during the study period. The regular practice of physical exercise seems to be related to a greater concern for health. In this sense, and given that we measured the practice of physical exercise before statin prescription, we could think that people with a high CVD risk but interested in their health status could try to compensate for this risk by carrying out more physical exercise. When this first attempt failed, they would receive high-intensity statin therapy. On the contrary, when the level of physical exercise is measured in patients who are already taking statins, the opposite effect could be observed, that is, subjects with a more intensive treatment would show a relaxed attitude towards physical exercise as they consider themselves protected by drugs.

Finally, the role of the known CVD risk factors in predicting the prescribing of high-intensity statins was lower than expected. This finding suggests that doctors often opt to prescribe high-intensity statins based on other unknown criteria, perhaps in part due to the lack of clear and direct indications in the European guidelines [1] on when to prescribe high-intensity statins, in contrast to current American College of Cardiology/American Heart Association (ACC/AHA) guidelines [2]. According to current European guidelines, in order to choose the type of statin to be prescribed, the doctor must know (i) the CVD risk of his patient, (ii) the LDL-C target levels that correspond to the patient according to his/her CVD risk, (iii) the percentage of LDL-C reduction that should be achieved to reach the LDL-C target levels, and (iv) the ability to reduce LDL-C levels of the different statins. This process, while accurate, can be confusing and costly, especially in the primary care setting, where time per patient is limited [25,26].

Our study has certain limitations. The homogeneity of the AWHS population makes it difficult to generalize the results, although the population is representative of a common group of statin users: middle-aged workers. In classifying of statins into high-intensity or low–moderate-intensity, we only took into account the type of statin prescribed. Usually however, this classification is made based on both the type of statin and the dose at which it is prescribed. Unfortunately, we had no data on the dose of the prescribed statin. Nonetheless, we believe that this limitation does not significantly affect the results obtained, as only high doses of atorvastatin and rosuvastatin can reduce LDL-C levels by more than 50%, and there is also a marked difference between the LDL-C-reducing capacity of rosuvastatin and atorvastatin at medium doses compared with the other drugs at their usual dosage [6]. Therefore, despite the potential existence of some bias in our study, this alone could not have accounted for the null association found. We also did not have information on other comorbidities and pharmacological treatments different from those studied, even though these could have influenced the decision to prescribe statins of greater or lesser intensity. Finally, as discussed in the methods section, certain treatments (e.g., beta-blockers) are not exclusively indicated for hypertension and may be prescribed for other diseases. Nonetheless, sensitivity analyses performed to assess possible bias yielded the same conclusions regardless of how hypertension was defined.

The strengths of this study include its prospective cohort nature, its large sample size (*n* > 600), and the inclusion of sociodemographic, clinical, lifestyle, and drug data. Furthermore, to our knowledge, ours is the first study to investigate the prescribing of statins and their intensity within the framework of the European guidelines for CVD prevention.

Our results show that in a significant proportion of individuals, the prescribing of statins and the type of statin prescribed for primary CVD prevention did not correspond with the indications in current guidelines. Therefore, it is necessary to develop new strategies to disseminate the guidelines, and to emphasize the differences in intensity between statins and the importance of reaching target LDL-C levels to prevent CVD [26], particularly in the context of the poor control of hypercholesterolemia and other CVD risk factors in the Spanish population [27,28]. It should also be noted that the intensity of the prescribed statin influences treatment persistence [29], and that higher-intensity statins such as simvastatin, atorvastatin, and rosuvastatin have been associated with greater diabetogenic effects [30]. Further research will be needed to help identify other as-yet-unknown factors that may influence the choice of statin intensity, such as availability, price, and industry pressure. Knowledge of these factors could enable the development of strategies to improve the clarity of the recommendations and their application in daily practice.

## 5. Conclusions

The results of this study show that the prescribing of high-intensity statins to new users for primary CVD prevention is partly independent of both basal CVD risk and the reduction in LDL-C required to reach recommended target levels. The probability of being prescribed high-intensity statins was higher in diabetic workers with high levels of LDL-C and in workers who performed more physical activity. Much of the variability in statin prescribing is due to as-yet-unknown factors.

## Figures and Tables

**Table 1:** Sociodemographic, clinical, and anthropometric characteristics of workers according to the intensity of statin prescribed.

		Statin Intensity		
	All (N = 683)	Low–Moderate-Intensity (N = 360)	High-Intensity (N = 323)	*p*
**Sociodemographic**				
**Mean (SD) age, y**	53.0 (4.0)	52.8 (4.3)	53.1 (3.7)	0.390
**Women, N (%)**	31 (4.5)	18 (5.0)	13 (4.0)	0.585
**Mean (SD) no. of years in factory**	28.4 (4.5)	28.4 (4.9)	28.4 (4.1)	0.963
**Work shift, N (%)**				0.137
Rotating	522 (76.5)	283 (78.8)	239 (74.0)	
Fixed	160 (23.4)	76 (21.2)	84 (26.0)	
**Work type, N (%)**				0.117
Assembly line/manual	590 (86.4)	318 (88.3)	272 (84.2)	
Sedentary	93 (13.6)	42 (11.7)	51 (15.8)	
**Clinical and anthropometric**				
**BMI (kg/m^2^), N (%)**				0.600
<25	127 (18.6)	67 (18.6)	60 (18.6)	
25–30	392 (57.4)	212 (58.9)	180 (55.7)	
≥30	164 (24.0)	81 (22.5)	83 (25.7)	
**EGFR < 60 mL/min/1.73 m^2^, N (%)**	8 (1.2)	6 (1.7)	2 (0.6)	0.292
**Total cholesterol (mg/dL), mean (SD)**	246.9 (37.5)	244.8 (35.0)	249.2 (40.0)	0.124
**LDL-cholesterol (mg/dL), mean (SD)**	159.0 (33.2)	157.7 (30.5)	160.6 (36.0)	0.258
**HDL-cholesterol (mg/dL), mean (SD)**	52.2 (11.5)	52.2 (11.4)	52.2 (11.6)	0.972
**Triglycerides (mg/dL), mean (SD)**	187.8 (139.7)	185.1 (148.5)	190.7 (129.4)	0.601
**Diabetes, N (%)**	69 (10.1)	33 (9.2)	36 (11.2)	0.392
**Hypertension, N (%)**	354 (51.8)	175 (48.6)	179 (55.4)	0.075
**Rheumatoid arthritis, N (%)**	12 (1.8)	5 (1.4)	7 (2.2)	0.563
**Previous TIA, N (%)**	3 (0.4)	2 (0.6)	1 (0.3)	1.000
**Arrhythmia, N (%)**	15 (2.2)	7 (1.9)	8 (2.5)	0.795

Abbreviations: BMI, body mass index; EGFR, estimated glomerular filtration rate; HDL, high-density lipoprotein; LDL, low-density lipoprotein; N, number; SD, standard deviation; TIA, transient ischemic attack. *p*-values correspond to t-test/chi-squared test or Fisher’s exact test.

**Table 2:** Lifestyle characteristics and subclinical atherosclerosis among workers, stratified by statin intensity.

		Statin Intensity		
	All (N = 683)	Low–Moderate-Intensity (N = 360)	High-Intensity (N = 323)	*p*
**Lifestyle**				
**Smoking, N (%)**				0.678
Never	131 (19.2)	73 (20.3)	58 (18.0)	
Current	249 (36.5)	127 (35.3)	122 (37.8)	
Former	302 (44.3)	160 (44.4)	142 (44.0)	
**Alcohol intake, N (%)**				0.685
Low risk (<40 g/d)	581 (85.1)	306 (85.0)	275 (85.1)	
Medium risk (40–60 g/d)	64 (9.4)	36 (10.0)	28 (8.7)	
High risk (>60 g/d)	38 (5.6)	18 (5.0)	20 (6.2)	
**Mediterranean diet adherence, N (%)**				0.379
Low	244 (35.7)	131 (36.4)	113 (35.0)	
Medium	372 (54.5)	189 (52.5)	183 (56.7)	
High	67 (9.8)	40 (11.1)	27 (8.4)	
**METs h/wk, mean (SD)**	31.0 (21.2)	29.6 (20.4)	32.6 (22.0)	0.065
**Sitting 5.5 h/day or more, N (%)**	361 (53.1)	189 (52.5)	172 (53.8)	0.744
**Sleep < 6 h/night, N (%)**	136 (20.0)	64 (17.8)	72 (22.4)	0.130
**Carotid atherosclerosis, N (%)**	273 (44.0)	130 (40.1)	143 (48.3)	**0.040**
**Femoral atherosclerosis, N (%)**	404 (65.6)	204 (61.1)	200 (70.9)	**0.010**

Abbreviations: METs h/wk, Metabolic equivalents of task hours per week; N, number; SD, standard deviation. *p*-values correspond to t-test/chi-squared test. Mediterranean diet adherence: Alternate Mediterranean Dietary Index (aMED) score. Low: 0–3 score in aMed Index. Medium: 4–6 score in aMed Index. High: 7–9 score in aMed Index. Statistically significant differences (*p* < 0.05) are shown in bold.

**Table 3:** Association between sociodemographic, analytical, clinical, and lifestyle factors and the prescribing of a high-intensity statin in the study population: logistic regression analyses (N = 569).

	Unadjusted Model	Multivariable Model
	OR (95%CI)	OR (95%CI)
**Age (years)**		
<50	0.90 (0.58–1.39)	1.03 (0.65–1.62)
50–55	1.00	1.00
55–60	0.88 (0.60–1.29)	0.90 (0.60–1.34)
>60	0.72 (0.25–2.09)	0.63 (0.21–1.90)
**LDL-cholesterol (mg/dL)**		
*Non-diabetics*		
<155	1.00	1.00
≥155	1.04 (0.73–1.49)	1.03 (0.72–1.49)
*Diabetics*		
<155	1.00	1.00
≥155	**4.50 (1.19–16.99)**	**4.96 (1.29–19.10)**
**Hypertension**		
No	1.00	1.00
Yes	1.25 (0.90–1.74)	1.26 (0.89–1.79)
**Diabetes**		
No	1.00	1.00
Yes	1.42 (0.78–2.56)	1.45 (0.78–2.69)
**Smoking**		
Never	1.00	1.00
Current	1.41 (0.88–2.25)	1.24 (0.74–2.06)
Former	1.20 (0.76–1.90)	1.13 (0.70–1.83)
**METs h/wk**		
<20	1.00	1.00
20–40	0.90 (0.60–1.35)	0.93 (0.62–1.41)
**>40**	**1.55 (1.03–2.32)**	**1.65 (1.08–2.50)**
**Carotid atherosclerosis**		
No	1.00	1.00
Yes	1.33 (0.96–1.86)	1.21 (0.85–1.71)
**Femoral atherosclerosis**		
No	1.00	1.00
Yes	**1.53 (1.08–2.17)**	1.45 (0.98–2.13)

Unadjusted Model: logistic regression model considering high-intensity statin as outcome and each individual variable as exposure. Adjusted Model: logistic regression model adjusted for age, smoking habit, LDL-cholesterol, diabetes, hypertension, METs h/wk, and atherosclerosis. Abbreviations: OR, odds ratio; 95%CI, 95% confidence interval; LDL-cholesterol, low-density lipoprotein cholesterol; METs h/wk, Metabolic equivalents of task hours per week. Statistically significant differences (*p* < 0.05) are shown in bold.

## References

[1] Piepoli M.F., Hoes A.W., Agewall S., Albus C., Brotons C., Catapano A.L., Cooney M.T., Corrà U., Cosyns B., Deaton C. (2016). 2016 European Guidelines on cardiovascular disease prevention in clinical practice. Eur. Heart J..

[2] Grundy S.M., Stone N.J., Bailey A.L., Beam C., Birtcher K.K., Blumenthal R.S., Braun L.T., de Ferranti S., Faiella-Tommasino J., Forman D.E. (2019). 2018 AHA/ACC/AACVPR/AAPA/ABC/ACPM/ADA/AGS/APhA/ASPC/NLA/PCNA Guideline on the Management of Blood Cholesterol: A Report of the American College of Cardiology/American Heart Association Task Force on Clinical Practice Guidelines. J. Am. Coll. Cardiol..

[3] Taylor F., Huffman M.D., Macedo A.F., Moore T.H.M., Burke M., Smith G.D., Ward K., Ebrahim S. (2013). Statins for the primary prevention of cardiovascular disease. Cochrane Database Syst. Rev..

[4] Mihaylova B., Emberson J., Blackwell L., Keech A., Simes J., Barnes E.H., Voysey M., Gray A., Collins R., Baigent C. (2012). The effects of lowering LDL cholesterol with statin therapy in people at low risk of vascular disease: Meta-analysis of individual data from 27 randomised trials. Lancet.

[5] Sniderman A., Thanassoulis G., Couture P., Williams K., Alam A., Furberg C.D. (2012). Is lower and lower better and better? A re-evaluation of the evidence from the Cholesterol Treatment Trialists’ Collaboration meta-analysis for low-density lipoprotein lowering. J. Clin. Lipidol..

[6] Law M.R., Wald N.J., Rudnicka A.R. (2003). Quantifying effect of statins on low density lipoprotein cholesterol, ischaemic heart disease, and stroke: Systematic review and meta-analysis. Br. Med. J..

[7] National Institute for Health Care Excellence (2016). Cardiovascular Disease: Risk Assessment and Reduction, Including Lipid Modification.

[8] Johal S., Jamsen K.M., Bell J.S., Mc Namara K.P., Magliano D.J., Liew D., Ryan-Atwood T.E., Anderson C., Ilomäki J. (2017). Do statin users adhere to a healthy diet and lifestyle? The Australian diabetes, obesity and lifestyle study. Eur. J. Prev. Cardiol..

[9] Sidell M.A., Ghai N.R., Reynolds K., Jacobsen S.J., Scott R., Van Den Eeden S., Caan B., Quinn V.P. (2019). Statins as a free pass: Body mass index and other cardiovascular risk factors among lipid-lowering medication users and nonusers in the California Men’s Health Study. Prev. Med..

[10] Macías Saint-Gerons D., De La Fuente Honrubia C., Montero Corominas D., Gil M.J., De Andrés-Trelles F., Catalá-López F. (2014). Standard and intensive lipid-lowering therapy with statins for the primary prevention of vascular diseases: A population-based study. Eur. J. Clin. Pharmacol..

[11] Ho K., Jamsen K.M., Bell J.S., Korhonen M.J., Mc Namara K.P., Magliano D.J., Liew D., Ryan-Atwood T.E., Shaw J.E., Luc S. (2018). Demographic, clinical and lifestyle factors associated with high-intensity statin therapy in Australia: The AusDiab study. Eur. J. Clin. Pharmacol..

[12] Casasnovas J.A., Alcaide V., Civeira F., Guallar E., Ibañez B., Borreguero J.J., Laclaustra M., León M., Peñalvo J.L., Ordovás J.M. (2012). Aragon workers’ health study—Design and cohort description. BMC Cardiovasc. Disord..

[13] Martin-Moreno J.M., Boyle P., Gorgojo L., Maisonneuve P., Fernandez-Rodriguez J.C., Salvini S., Willett W.C. (1993). Development and validation of a food frequency questionnaire in Spain. Int. J. Epidemiol..

[14] Martínez-González M.A., López-Fontana C., Varo J.J., Sánchez-Villegas A., Martinez J.A. (2005). Validation of the Spanish version of the physical activity questionnaire used in the Nurses’ Health Study and the Health Professionals’ Follow-up Study. Public Health Nutr..

[15] Laclaustra M., Casasnovas J.A., Fernández-Ortiz A., Fuster V., León-Latre M., Jiménez-Borreguero L.J., Pocovi M., Hurtado-Roca Y., Ordovas J.M., Jarauta E. (2016). Femoral and Carotid Subclinical Atherosclerosis Association with Risk Factors and Coronary Calcium: The AWHS Study. J. Am. Coll. Cardiol..

[16] Levey A.S., Bosch J.P., Lewis J.B., Greene T., Rogers N., Roth D. (1999). A more accurate method to estimate glomerular filtration rate from serum creatinine: A new prediction equation. Modification of Diet in Renal Disease Study Group. Ann. Intern. Med..

[17] Levey A.S., Coresh J., Balk E., Kausz A.T., Levin A., Steffes M.W., Hogg R.J., Perrone R.D., Lau J., Eknoyan G. (2003). National Kidney Foundation practice guidelines for chronic kidney disease: Evaluation, classification, and stratification. Ann. Intern. Med..

[18] Friedewald W.T., Levy R.I., Fredrickson D.S. (1972). Estimation of the Concentration of Low-Density Lipoprotein Cholesterol in Plasma, Without Use of the Preparative Ultracentrifuge. Clin. Chem..

[19] Fung T.T., Rexrode K.M., Mantzoros C.S., Manson J.E., Willett W.C., Hu F.B. (2009). Mediterranean diet and incidence of and mortality from coronary heart disease and stroke in women. Circulation.

[20] Ainsworth B.E., Haskell W.L., Herrmann S.D., Meckes N., Bassett D.R., Tudor-Locke C., Greer J.L., Vezina J., Whitt-Glover M.C., Leon A.S. (2011). 2011 compendium of physical activities: A second update of codes and MET values. Med. Sci. Sports Exerc..

[21] León-Latre M., Moreno-Franco B., Andrés-Esteban E.M., Ledesma M., Laclaustra M., Alcalde V., Peñalvo J.L., Ordovás J.M., Casasnovas J.A. (2014). Sedentarismo y su relación con el perfil de riesgo cardiovascular, la resistencia a la insulina y la inflamación. Rev. Esp. Cardiol..

[22] Hirshkowitz M., Whiton K., Albert S.M., Alessi C., Bruni O., DonCarlos L., Hazen N., Herman J., Katz E.S., Kheirandish-Gozal L. (2015). National sleep foundation’s sleep time duration recommendations: Methodology and results summary. Sleep Health.

[23] Conroy R.M., Pyörälä K., Fitzgerald A.P., Sans S., Menotti A., De Backer G., De Bacquer D., Ducimetière P., Jousilahti P., Keil U. (2003). Estimation of ten-year risk of fatal cardiovascular disease in Europe: The SCORE project. Eur. Heart J..

[24] Sarwar N., Gao P., Kondapally Seshasai S.R., Gobin R., Kaptoge S., Di Angelantonio E., Ingelsson E., Lawlor D.A., Selvin E., Stampfer M. (2010). Diabetes mellitus, fasting blood glucose concentration, and risk of vascular disease: A collaborative meta-analysis of 102 prospective studies. Lancet.

[25] Mira J.J., Nebot C., Lorenzo S., Pérez-Jover V. (2010). Patient report on information given, consultation time and safety in primary care. Qual. Saf. Health Care.

[26] Milà L., Barrabés J.A., Lidón R.M., Sambola A., Bañeras J., Oristrell G., Rafecas A., García-Dorado D. (2019). Prior adherence to recommended lipid control targets in patients admitted for acute coronary syndrome. Rev. Esp. Cardiol..

[27] Guallar-Castillón P., Gil-Montero M., León-Muñoz L.M., Graciani A., Bayán-Bravo A., Taboada J.M., Banegas J.R., Rodríguez-Artalejo F. (2012). Magnitude and management of hypercholesterolemia in the adult population of Spain, 2008–2010: The ENRICA study. Rev. Esp. Cardiol..

[28] Aguilar-Palacio I., Malo S., Feja C., Lallana M.J., León-Latre M., Casasnovas J.A., Rabanaque M., Guallar E. (2018). Risk factors control for primary prevention of cardiovascular disease in men: Evidence from the Aragon Workers Health Study (AWHS). PLoS ONE.

[29] Malo S., Aguilar-Palacio I., Feja C., Menditto E., Lallana M.J., Andrade E., Casasnovas J.A., Rabanaque M.J. (2018). Persistence With Statins in Primary Prevention of Cardiovascular Disease: Findings From a Cohort of Spanish Workers. Rev. Esp. Cardiol..

[30] Laakso M., Kuusisto J. (2017). Diabetes Secondary to Treatment with Statins. Curr. Diabetes Rep..

